# NanoOK: multi-reference alignment analysis of nanopore sequencing data, quality and error profiles

**DOI:** 10.1093/bioinformatics/btv540

**Published:** 2015-09-17

**Authors:** Richard M. Leggett, Darren Heavens, Mario Caccamo, Matthew D. Clark, Robert P. Davey

**Affiliations:** The Genome Analysis Centre (TGAC), Norwich NR4 7UH, UK

## Abstract

**Motivation:** The Oxford Nanopore MinION sequencer, currently in pre-release testing through the MinION Access Programme (MAP), promises long reads in real-time from an inexpensive, compact, USB device. Tools have been released to extract FASTA/Q from the MinION base calling output and to provide basic yield statistics. However, no single tool yet exists to provide comprehensive alignment-based quality control and error profile analysis—something that is extremely important given the speed with which the platform is evolving.

**Results:** NanoOK generates detailed tabular and graphical output plus an in-depth multi-page PDF report including error profile, quality and yield data. NanoOK is multi-reference, enabling detailed analysis of metagenomic or multiplexed samples. Four popular Nanopore aligners are supported and it is easily extensible to include others.

**Availability and implementation**: NanoOK is an open-source software, implemented in Java with supporting R scripts. It has been tested on Linux and Mac OS X and can be downloaded from https://github.com/TGAC/NanoOK. A VirtualBox VM containing all dependencies and the DH10B read set used in this article is available from http://opendata.tgac.ac.uk/nanook/. A Docker image is also available from Docker Hub—see program documentation https://documentation.tgac.ac.uk/display/NANOOK.

**Contact:** richard.leggett@tgac.ac.uk

**Supplementary information:** Supplementary data are available at *Bioinformatics* online.

## 1 Introduction

The Oxford Nanopore Technologies (ONT) MinION is one of the latest of a new generation of single molecule sequencing technologies. Originally revealed at the 2012 Advances in Genome Biology and Technology conference, it arrived in the labs of MinION Access Programme (MAP) members in May 2014. Offering multi-kilobase reads, the MinION attracted interest due to its compact size, USB connection, relatively inexpensive expected purchase price and a streamed mode of operation that enables analysis of data as it generated. Though not out of pre-release testing, the device is in the hands of research groups around the world who are evaluating the performance and suitability of the platform for a wide range of applications including medical diagnostics, environmental surveillance and *de novo* sequencing. ONT’s technology involves the detection of current changes across biological nanopores through which DNA molecules move. The degree of current change depends on the bases (5-mer) present in the pore at any given time and multiple measurements are made as the molecule advances, resulting in a characteristic ‘squiggle’ plot which ONT’s basecalling software (Metrichor) processes to a nucleotide sequence. The MinION will read both strands of DNA (Template and Complement) and the software will attempt to call a consensus 2D (2-directional) read. Individual reads (one per file) are output in FAST5 format, an implementation of the HDF5 standard. Two tools—poretools (Loman and Quinlan, 2014) and poRe (Watson *et al.,* 2014)—have already been published to extract reads to FASTA or FASTQ format and to plot graphs of yield, read size and pore occupancy. Within the MAP community, the most popular aligners have emerged as LAST (Kielbasa *et al.,* 2011), BLASR (Chaisson and Tesler, 2012) and BWA-MEM (Li, 2013), which introduced an ‘ont2d’ option with version 0.7.11. A Nanopore-specific aligner, marginAlign (Jain *et al.,* 2015), has also been developed which begins with guide alignments produced by BLASR, BWA-MEM, LAST or LASTZ and produces a trained realignment based on a model of Nanopore error profiles. After alignment, individual labs tend to use their own *ad* *hoc* approaches for analysing data, with no published tools available to provide detailed post-alignment analysis. A web-based application for monitoring MinION runs, minoTour (http://minotour.nottingham.ac.uk), has been developed which provides a wide range of very useful QC metrics in real-time. As reads emerge, minoTour will align them against a reference but this analysis is currently limited to coverage assessment and variant calling. Without a comprehensive post-alignment tool, analysis can be unnecessarily time consuming and can require specialized programming skills. NanoOK is designed to address this need, providing quick and intuitive alignment-based analysis and quality control of Nanopore runs, facilitating comparison across chemistry, flow cell, base calling changes and alignment tools. NanoOK itself extracts FASTA files, but users can also use third party tools. NanoOK carries out alignments via a number of supported alignment tools and will produce tabular output files from which it also generates graphs and a multi-page PDF report.

## 2 Methods

NanoOK expects runs to be organized in sample directories—initially these contain the reads output by the Metrichor software, but NanoOK will add further subdirectories containing analysis and output. There are three simple steps involved in running NanoOK. Firstly, FASTA or FASTQ reads are extracted:

nanook extract -s MyNanoporeRun -a

Alignments are initiated with:

nanook align -s MyNanoporeRun -r refname.fa

Finally, alignments are analysed and plain text machine readable analysis files, graphs and PDF report are generated with:

nanook analyse -s MyNanoporeRun -r refname.fa

NanoOK will create further subdirectories for FASTA or FASTQ files, alignments, analysis files, logs and LaTeX files as it runs.

When executing the analysis phase, NanoOK performs the following sequence of actions:

1. Read reference FASTA file and store IDs and lengths.
2. For each set of reads (Template, Complement and 2D) within pass and fail directories, store length of each read.
3. For each set of reads (Template, Complement and 2D) within the pass and fail directories, parse the alignment and store details of errors, quality and accuracy.
4. Write a set of analysis files—tabbed plain text files suitable for machine reading or graph plotting.
5. Initiate graph plotting through the R environment.
6. Build a LaTeX report file and then PDF with pdflatex.

NanoOK currently supports four alignment tools—LAST (the default), BWA-MEM, BLASR and marginAlign. However, NanoOK facilitates the addition of new parsers by implementing Java classes supporting an AlignmentFileParser interface. The basis for NanoOK’s analysis of sequencing errors (substitutions, insertions, deletions, perfect sequence, error motifs) is base-by-base parsing of the alignment strings found in the MAF format files used by LAST. BWA, BLASR and marginAlign output SAM format files, so NanoOK uses a CIGARString class to convert the CIGAR-format alignment strings in SAM files into MAF style strings.

An individual read may produce multiple alignments to one or more references. In this instance, NanoOK takes the highest scoring alignment and merges this with any alignments for the same reference that map close to the initial alignment. Merging is performed by expanding the co-ordinates of the original alignment to include the nearby alignment; analysis of matches, mismatches and indels is then performed on the combined alignment. In the interests of execution speed, NanoOK supports multithreading at all stages. Users can specify threads using a –t parameter.

## 3 Results

The Supplementary material contains an example NanoOK report for a run of *Escherichia coli* K12 substr. DH10B produced using an R7.3 flow cell. The report contains data on the whole run, as well as separate sections for each reference (in this case, control sequence and *E. coli*). At the run level, data include:

- Pre-alignment summary—including pass/fail counts and read length distributions for Template, Complement and 2D reads.
- Alignment summary—including counts of reads aligning to each reference, mean length, aligned bases, coverage.
- Substitution analysis—like Jain *et al.* (2015), we observe smaller percentage of A to T and T to A substitutions.
- Error kmers—3/4/5-mers occuring before substitutions, insertions and deletions, along with error motif images. Here, we find a high abundance of low complexity kmers, indicative of homopolymer problems, as observed in Jain *et al.* (2015).

For each reference:

- Error analysis—identity, insertions, deletions, substitutions.
- Identity—read identity histograms, scatter plots of read identity versus length, alignment identity versus percent of read aligned, percentage of read aligned versus length.
- Coverage—coverage of reference in Template, Complement and 2D reads and GC content of reference.
- Perfect kmers—analysis of longest perfect sequence without any errors compared with reference.
- Over- and under-represented kmers—tables and scatter plots of 5-mers in reference versus 5-mers in reads.

## 4 Summary

NanoOK provides comprehensive alignment-based analysis of Nanopore reads through a simple, easy to use interface. During our progress through the MAP, we have found it to be an invaluable tool for understanding the data emerging from the sequencer as the platform evolves and we believe it will have wide applicability to other groups working with ONT’s technology. The speed of change within the MAP is rapid and requires a tool such as NanoOK to evaluate changes: at the time of writing there have been four flowcell versions, five genomic sequencing kits, multiple versions of sequencer operating software and base caller, and at the recent London Calling meeting ONT announced forthcoming new MinIONs (MK1), flowcell chemistries (R8.0) and a new fast run mode.

## Supplementary Material

Supplementary Data
