# Supplementary material for: NanoOK: multi-reference alignment analysis of nanopore sequencing data, quality and error profiles
Source: Bioinformatics. 2015 Sep 17;32(1):142–4. doi: 10.1093/bioinformatics/btv540 (PMC4681994; doi:10.1093/bioinformatics/btv540)
Supplement: Supplementary Data [file supp_32_1_142__index.html]

NanoOK: Multi-reference alignment analysis of nanopore sequencing data, quality and error profiles — NanoOK: multi-reference alignment analysis of nanopore sequencing data, quality and error profiles — NanoOK: multi-reference alignment analysis of nanopore sequencing data, quality and error profiles — Supplementary Data 

# NanoOK: multi-reference alignment analysis of nanopore sequencing data, quality and error profiles

## Supplementary Data

files

- Supplementary Data - pdf file
