# Supplementary material for: NanoOK: multi-reference alignment analysis of nanopore sequencing data, quality and error profiles
Source: Bioinformatics. 2015 Sep 17;32(1):142–4. doi: 10.1093/bioinformatics/btv540 (PMC4681994; doi:10.1093/bioinformatics/btv540)
Supplement: Supplementary Data [file supp_btv540_N79596_dh10b_8kb_11022015.pdf]

NanoOK report for N79596_dh10b_8kb_11022015

Pass and fail counts

Type	Pass	Fail
Template	4418	0
Complement	4418	0
2D	4418	0

Read lengths

Type	NumReads	TotalBases	Mean	Longest	Shortest	N50	N50Count	N90	N90Count
Template	4418	14893365	3371.06	17659	207	4430	1250	2000	3116
Complement	4418	15814292	3579.51	18515	238	4667	1262	2124	3142
2D	4418	16154386	3656.49	19885	235	4807	1251	2178	3120

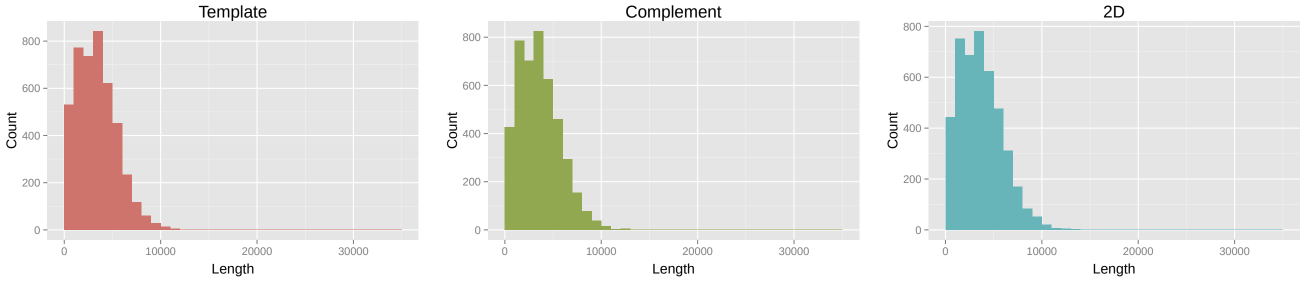

Template alignments

Number of reads	4418	
Number of reads with alignments	4113	(93.10%)
Number of reads without alignments	305	(6.90%)

ID	Size	Number of Reads	% of Reads	Mean read length	Aligned bases	Mean coverage	Longest Perf Kmer
DNA CS	3560	182	4.12	2942.81	594379	166.96	57
Escherichia coli	4686137	3931	88.98	3494.43	15267456	3.26	73

Complement alignments

Number of reads	4418	
Number of reads with alignments	4185	(94.73%)
Number of reads without alignments	233	(5.27%)

ID	Size	Number of Reads	% of Reads	Mean read length	Aligned bases	Mean coverage	Longest Perf Kmer
DNA CS	3560	183	4.14	3108.88	594507	167.00	49
Escherichia coli	4686137	4002	90.58	3665.31	15658978	3.34	66

2D alignments

Number of reads	4418	
Number of reads with alignments	4262	(96.47%)
Number of reads without alignments	156	(3.53%)

ID	Size	Number of Reads	% of Reads	Mean read length	Aligned bases	Mean coverage	Longest Perf Kmer
DNA CS	3560	186	4.21	3133.24	608745	171.00	138
Escherichia coli	4686137	4076	92.26	3695.06	15745543	3.36	187

DNA CS error analysis

	Template	Complement	2D
Overall base identity (excluding indels)	74.56%	70.60%	85.96%
Aligned base identity (excluding indels)	80.77%	80.86%	91.67%
Identical bases per 100 aligned bases (including indels)	67.19%	67.57%	82.30%
Inserted bases per 100 aligned bases (including indels)	3.43%	5.35%	4.17%
Deleted bases per 100 aligned bases (including indels)	13.38%	11.09%	6.05%
Substitutions per 100 aligned bases (including indels)	16.00%	15.99%	7.48%
Mean insertion size	1.59	1.64	1.55
Mean deletion size	1.67	1.66	1.55

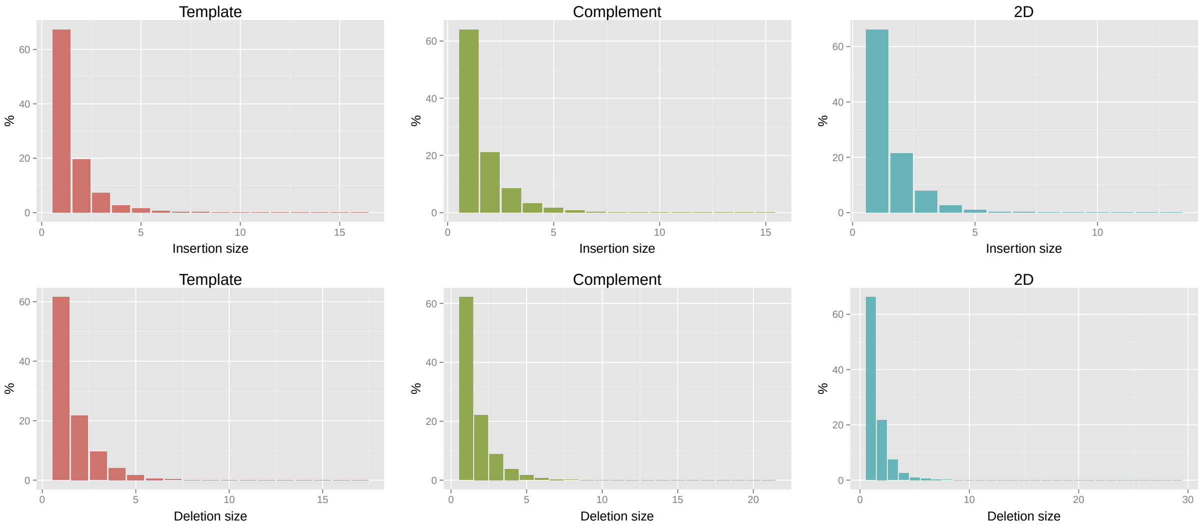

DNA CS read identity

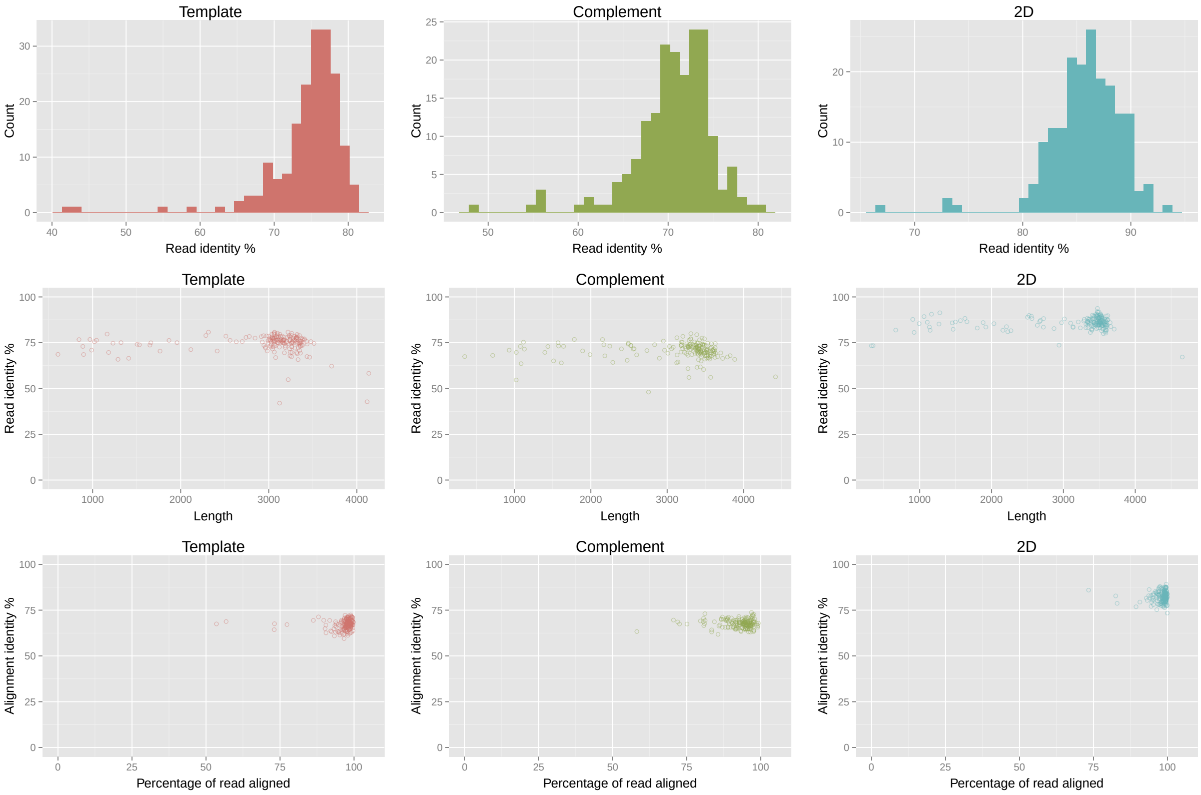

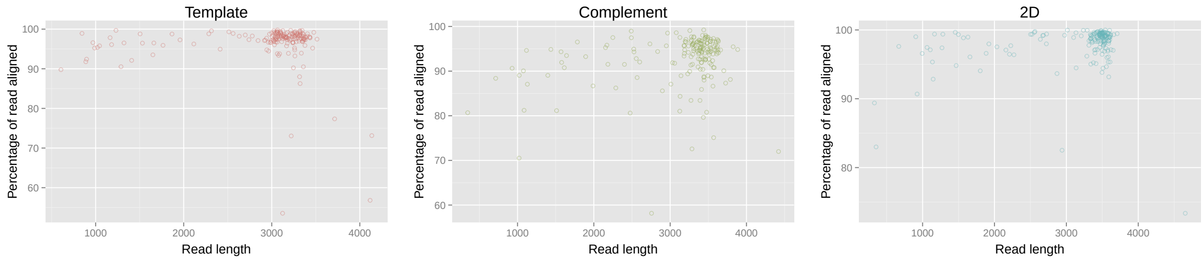

DNA CS perfect kmers

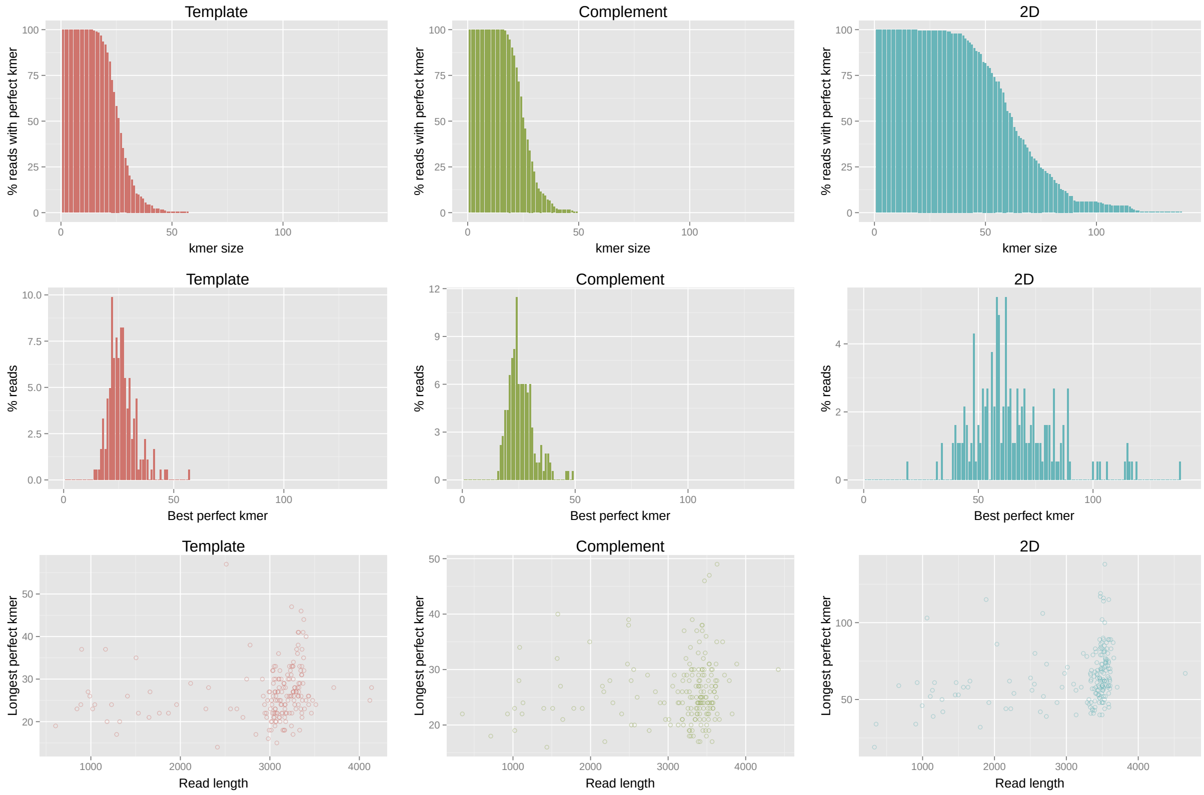

DNA CS coverage

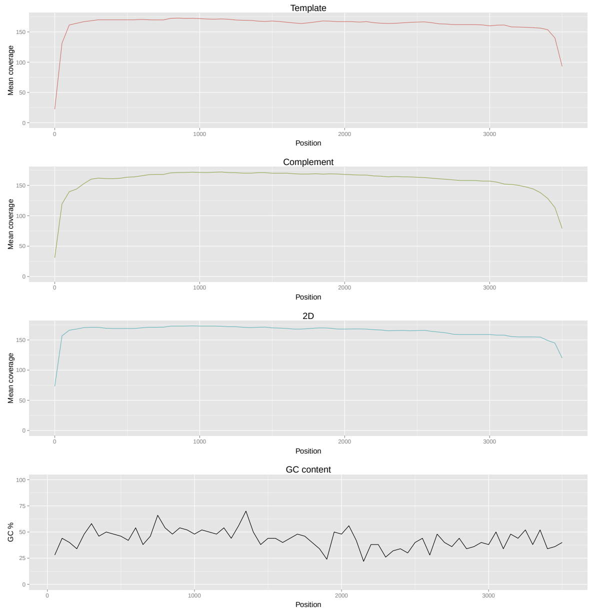

DNA CS 5-mer analysis

Under-represented 5-mers

	Template				Complement				2D			
Rank	kmer	Ref %	Read %	Diff %	kmer	Ref %	Read %	Diff %	kmer	Ref %	Read %	Diff %
1	TTTTT	0.759	0.079	-0.680	TTTTT	0.759	0.107	-0.651	TTTTT	0.759	0.045	-0.714
2	AAAAA	0.478	0.051	-0.427	AAAAA	0.478	0.075	-0.402	AAAAA	0.478	0.062	-0.416
3	AAAAC	0.337	0.099	-0.238	AAAAC	0.337	0.118	-0.219	TGATG	0.393	0.183	-0.210
4	TGATG	0.393	0.161	-0.233	GATGT	0.309	0.122	-0.187	GATGT	0.309	0.142	-0.167
5	AATAT	0.309	0.100	-0.209	TGATG	0.393	0.228	-0.166	CTTTT	0.253	0.101	-0.151
6	GATGT	0.309	0.101	-0.208	AACAA	0.281	0.122	-0.159	CTGAT	0.309	0.158	-0.151
7	GTTTT	0.281	0.114	-0.167	GCAAT	0.309	0.155	-0.154	AAAAC	0.337	0.188	-0.149
8	CTGAT	0.309	0.143	-0.166	GTCAG	0.281	0.134	-0.147	TTATC	0.309	0.177	-0.132
9	TAAAA	0.225	0.059	-0.166	AATAT	0.309	0.163	-0.146	TGTGA	0.225	0.093	-0.131
10	AGTAA	0.253	0.094	-0.158	AGTAA	0.253	0.107	-0.146	GCTGA	0.281	0.155	-0.126

Over-represented 5-mers

	Template				Complement				2D			
Rank	kmer	Ref %	Read %	Diff %	kmer	Ref %	Read %	Diff %	kmer	Ref %	Read %	Diff %
1	CTTTG	0.028	0.156	0.128	GAGGA	0.000	0.149	0.149	ATCAG	0.056	0.189	0.133
2	TCGGG	0.028	0.150	0.122	GAGAG	0.112	0.261	0.148	TCAGC	0.028	0.161	0.133
3	AACGT	0.084	0.198	0.114	GGAGA	0.028	0.148	0.120	CATCA	0.112	0.230	0.117
4	CATCT	0.000	0.113	0.113	TCAGC	0.028	0.142	0.114	GCATC	0.084	0.198	0.113
5	GCTCC	0.000	0.111	0.111	GTGTG	0.000	0.113	0.113	AACCA	0.028	0.140	0.111
6	TCTTA	0.000	0.111	0.111	TCGGG	0.028	0.141	0.113	CAGCA	0.056	0.164	0.107
7	TCAGC	0.028	0.140	0.111	GTATC	0.000	0.109	0.109	CATCT	0.000	0.103	0.103
8	GAGGA	0.000	0.111	0.111	TACTT	0.000	0.108	0.108	ACCAA	0.028	0.122	0.094
9	GGGGA	0.028	0.136	0.108	CGAGA	0.000	0.108	0.108	CTTTG	0.028	0.115	0.087
10	ATTAG	0.028	0.134	0.106	CTTTG	0.028	0.135	0.107	CAACA	0.084	0.166	0.082

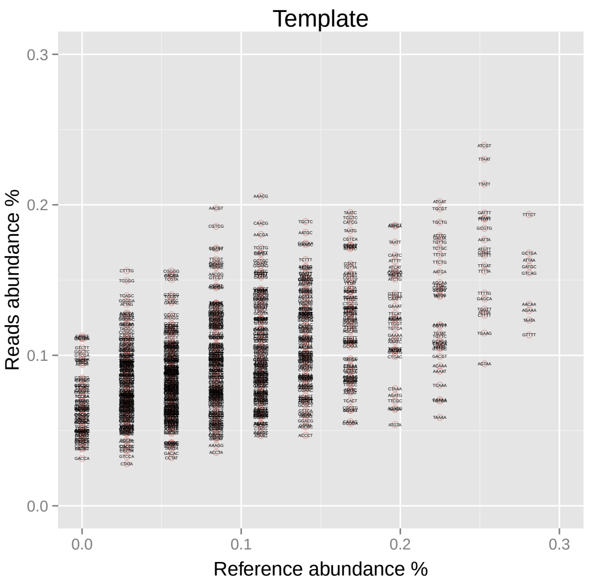

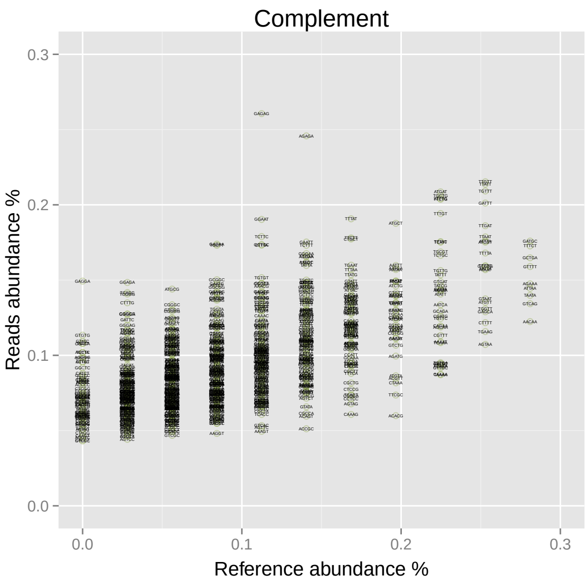

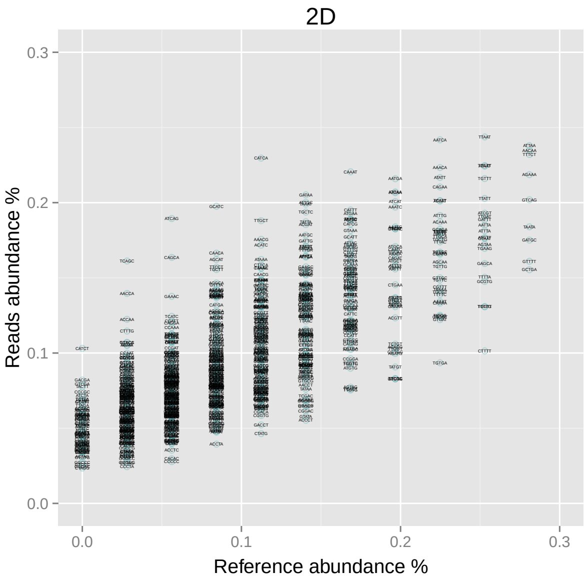

Escherichia coli error analysis

	Template	Complement	2D
Overall base identity (excluding indels)	74.27%	72.39%	86.02%
Aligned base identity (excluding indels)	80.42%	81.26%	91.68%
Identical bases per 100 aligned bases (including indels)	66.83%	67.81%	82.28%
Inserted bases per 100 aligned bases (including indels)	3.79%	5.58%	4.57%
Deleted bases per 100 aligned bases (including indels)	13.12%	10.98%	5.68%
Substitutions per 100 aligned bases (including indels)	16.27%	15.63%	7.47%
Mean insertion size	1.59	1.66	1.59
Mean deletion size	1.68	1.64	1.50

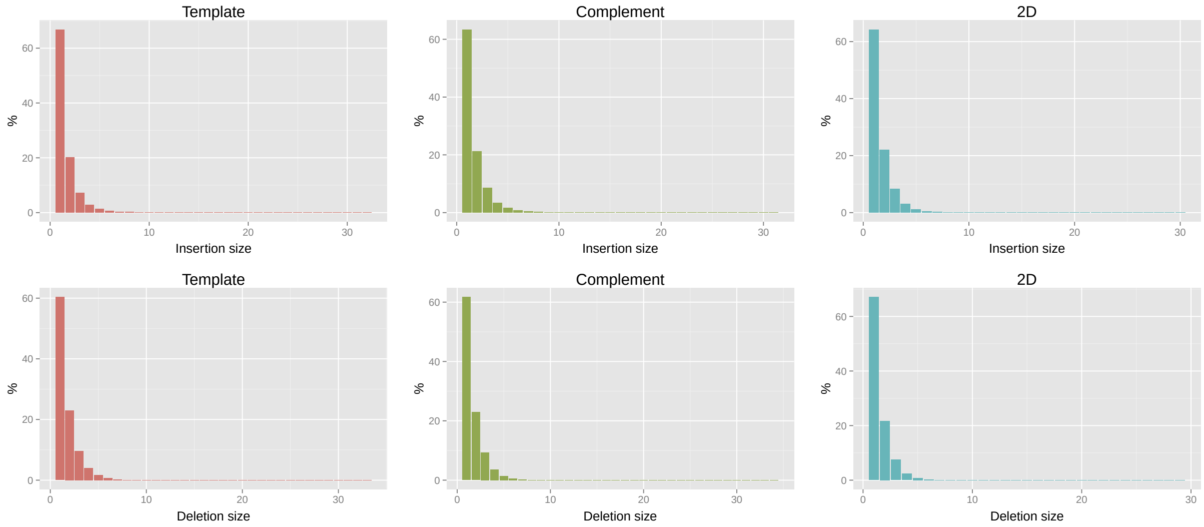

Escherichia coli read identity

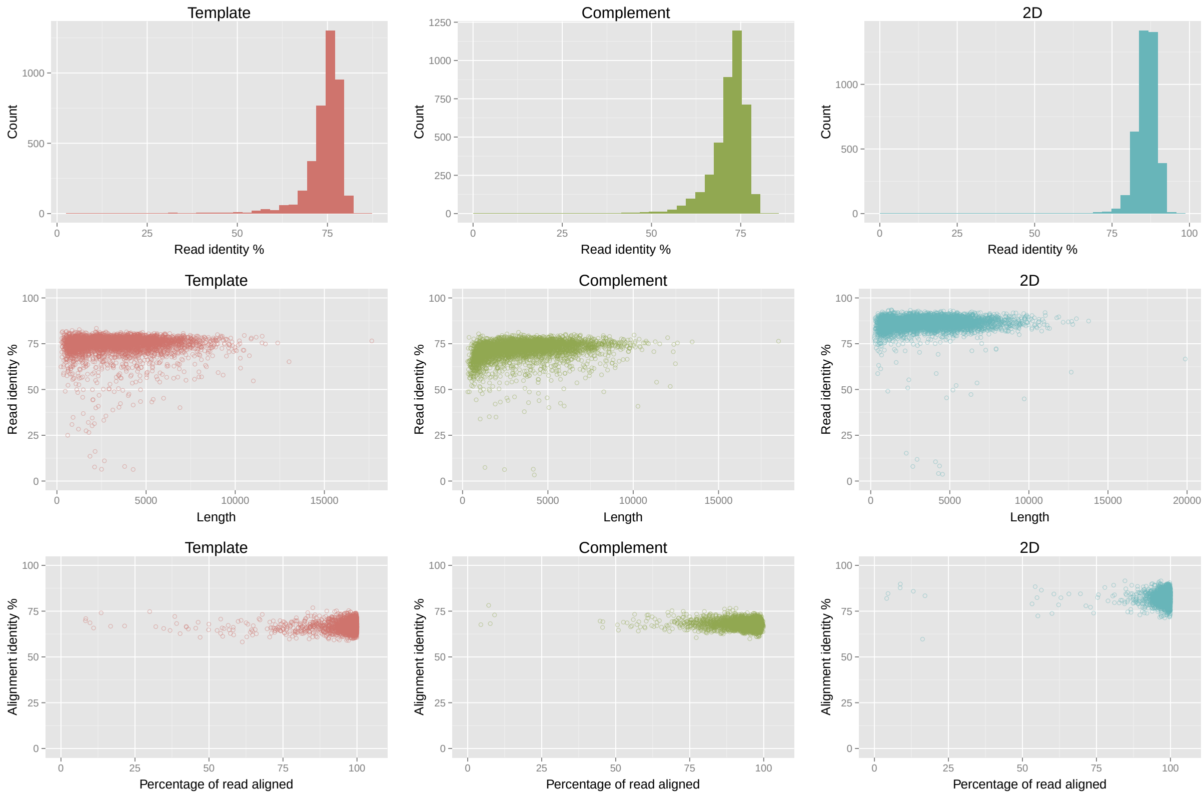

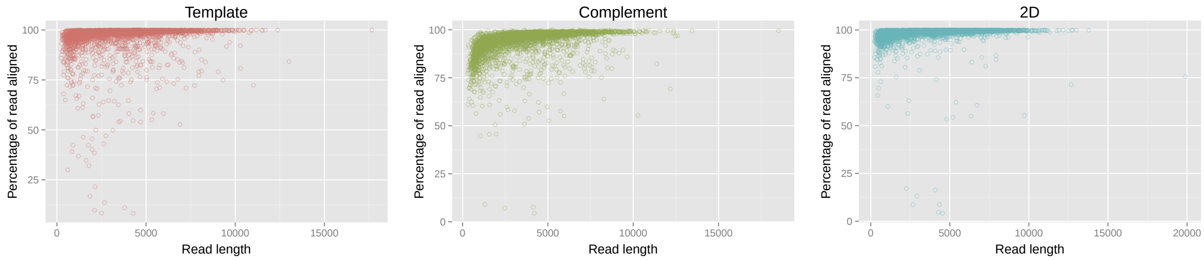

Escherichia coli perfect kmers

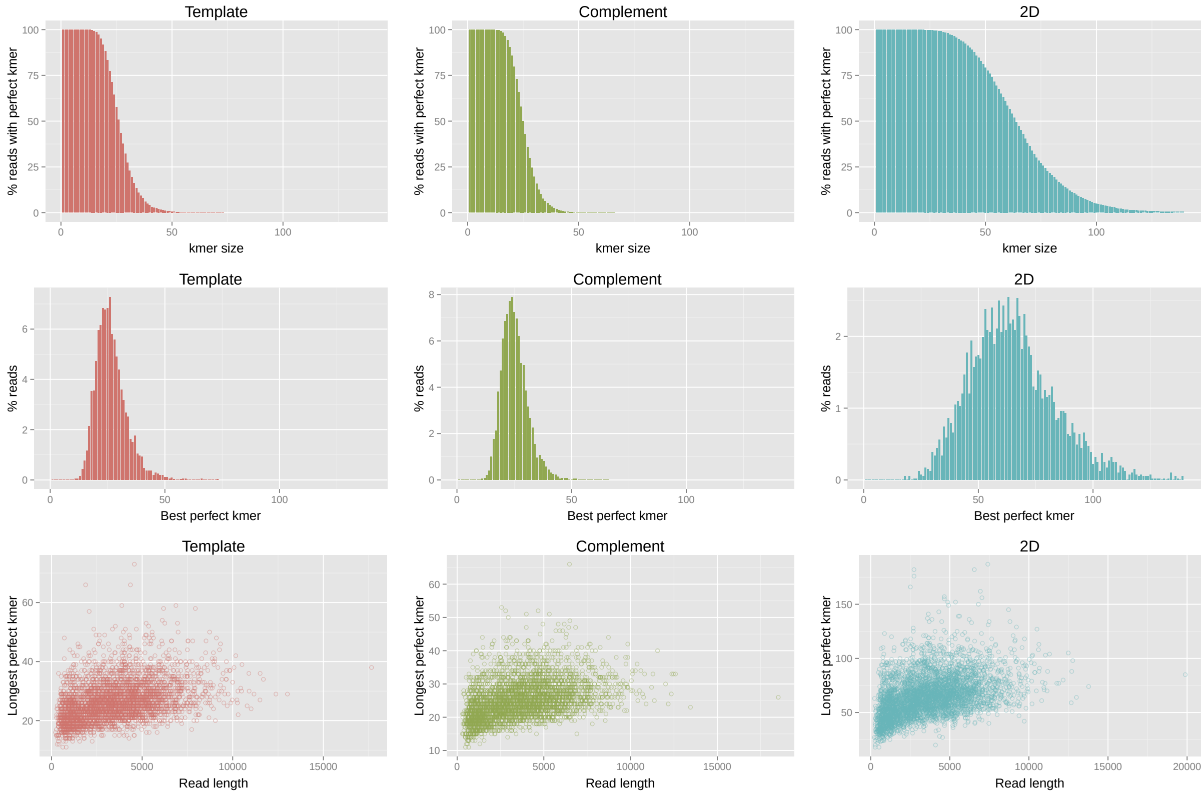

Escherichia coli coverage

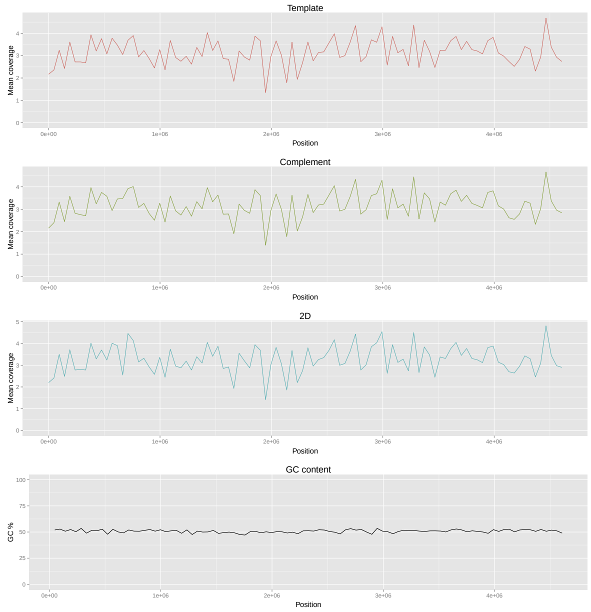

Escherichia coli 5-mer analysis

Under-represented 5-mers

	Template				Complement				2D			
Rank	kmer	Ref %	Read %	Diff %	kmer	Ref %	Read %	Diff %	kmer	Ref %	Read %	Diff %
1	AAAAA	0.246	0.049	-0.198	CGCCA	0.285	0.079	-0.206	TTTTT	0.251	0.035	-0.216
2	TTTTT	0.251	0.064	-0.187	AAAAA	0.246	0.065	-0.182	AAAAA	0.246	0.038	-0.209
3	CGCCA	0.285	0.106	-0.178	TTTTT	0.251	0.072	-0.178	CGCCA	0.285	0.185	-0.100
4	CGCTG	0.259	0.087	-0.172	GCCAG	0.279	0.128	-0.152	TGGCG	0.275	0.180	-0.095
5	GCCAG	0.279	0.131	-0.148	CCAGC	0.287	0.137	-0.150	GCCAG	0.279	0.192	-0.088
6	CGCCG	0.219	0.082	-0.137	CTGGC	0.279	0.135	-0.144	AAAAT	0.194	0.107	-0.087
7	CTGGC	0.279	0.151	-0.128	CAGCA	0.262	0.136	-0.126	CTGGC	0.279	0.198	-0.082
8	GCCGC	0.209	0.082	-0.127	TCGCC	0.203	0.079	-0.124	CAAAA	0.169	0.088	-0.081
9	ACGCC	0.176	0.054	-0.121	TGGCG	0.275	0.151	-0.123	GCTGG	0.279	0.201	-0.078
10	GCTGG	0.279	0.161	-0.119	CACCA	0.182	0.059	-0.123	TTTTG	0.172	0.099	-0.073

Over-represented 5-mers

	Template				Complement				2D			
Rank	kmer	Ref %	Read %	Diff %	kmer	Ref %	Read %	Diff %	kmer	Ref %	Read %	Diff %
1	CGGGG	0.055	0.162	0.107	GAGAG	0.045	0.238	0.193	CAAAT	0.105	0.157	0.053
2	GTCGT	0.078	0.172	0.094	AGAGA	0.071	0.220	0.149	AAGGA	0.056	0.097	0.041
3	TCGGG	0.059	0.152	0.092	CGGGG	0.055	0.155	0.100	CTCGT	0.043	0.080	0.037
4	TCGTA	0.053	0.137	0.085	GGGGG	0.031	0.129	0.097	AGGCA	0.093	0.129	0.035
5	CGTAG	0.058	0.142	0.084	GGGGC	0.060	0.153	0.093	TCTAG	0.004	0.039	0.035
6	GGGGC	0.060	0.141	0.081	CTGAG	0.050	0.135	0.085	TAAAT	0.112	0.147	0.035
7	TCGTC	0.094	0.172	0.078	GAGGG	0.031	0.109	0.078	CTAGC	0.008	0.041	0.033
8	CGTCG	0.114	0.190	0.076	GCTAG	0.008	0.086	0.078	GATTC	0.077	0.111	0.033
9	TAGGC	0.031	0.104	0.074	TCGGG	0.059	0.133	0.074	GAAGG	0.095	0.128	0.033
10	TTAGT	0.038	0.111	0.073	GAGAA	0.090	0.163	0.073	TTGGA	0.029	0.062	0.032

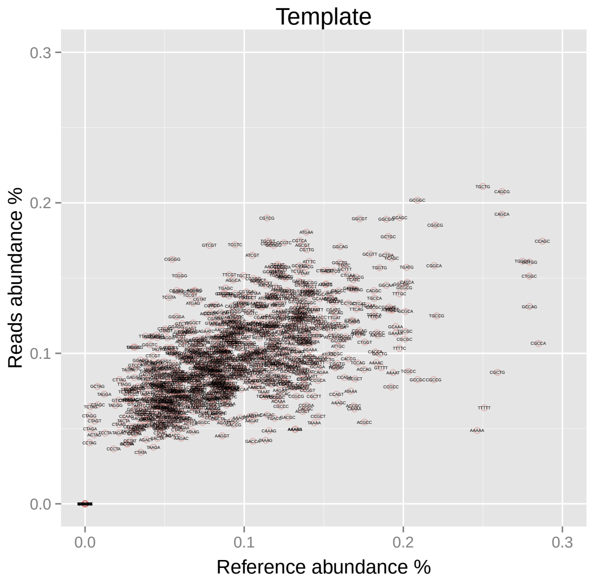

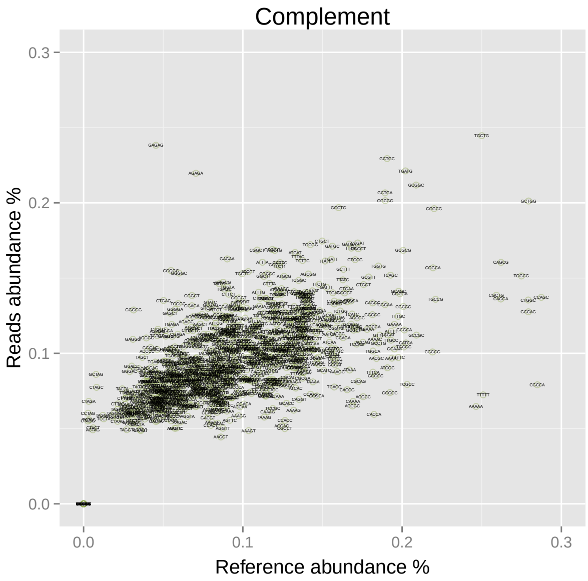

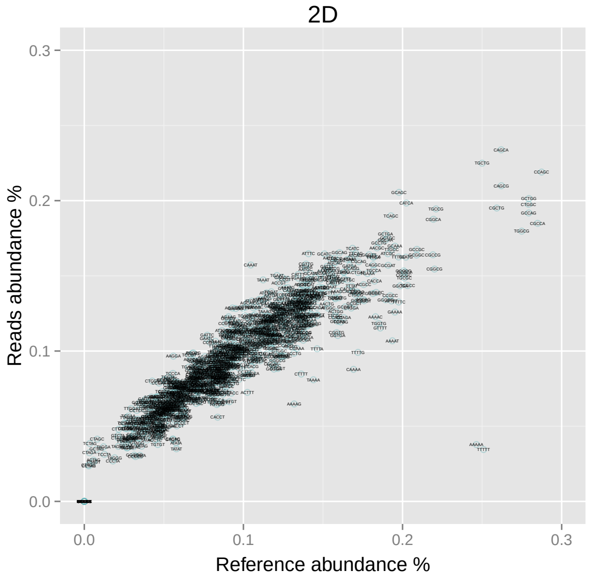

All reference 21mer analysis

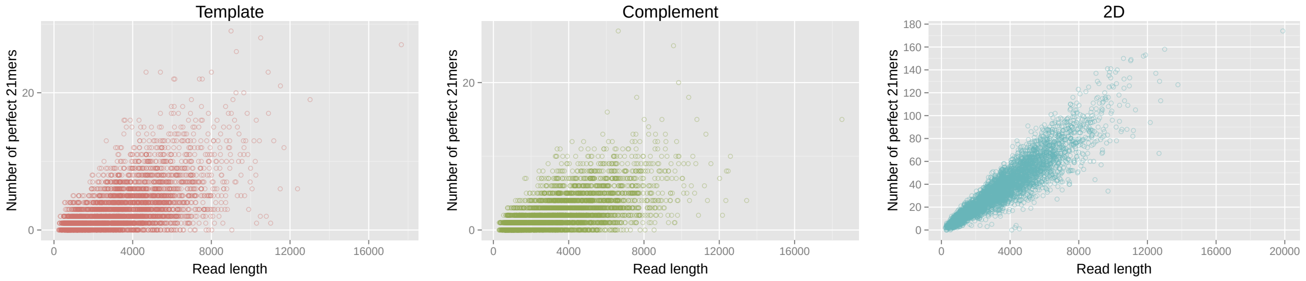

All reference substitutions

		Template substituted %				Complement substituted %				2D substituted %			
		a	c	g	t	a	c	g	t	a	c	g	t
Reference	A	0.00	8.59	9.63	4.98	0.00	8.98	9.13	5.28	0.00	8.42	8.36	4.58
	C	8.39	0.00	8.79	9.51	9.41	0.00	8.68	8.80	9.31	0.00	10.12	9.23
	G	9.23	8.85	0.00	8.51	8.93	8.52	0.00	9.20	9.25	10.00	0.00	9.45
	T	5.01	9.85	8.66	0.00	5.24	8.96	8.87	0.00	4.55	8.35	8.38	0.00

Kmer motifs before errors

3-mer error motif analysis

	Template			Complement			2D			
Rank	Insertion	Deletion	Substitution	Insertion	Deletion	Substitution	Insertion	Deletion	Substitution	
1	TTC (3.06%)	AAA (3.46%)	AAA (3.61%)	AAA (2.75%)	AAA (3.49%)	AAA (3.74%)	GCA (3.03%)	AAA (5.45%)	AAA (3.84%)	Most common
2	GCA (2.65%)	TTC (3.12%)	TTC (3.49%)	TGC (2.74%)	GAA (2.89%)	GCA (3.48%)	AAA (2.80%)	TTT (4.57%)	GCA (3.83%)	
3	AAA (2.65%)	TGC (2.79%)	GCA (3.27%)	GCA (2.63%)	TTT (2.73%)	GAA (3.27%)	TTC (2.69%)	GAA (2.59%)	GAA (3.34%)	
4	TGC (2.61%)	GCA (2.72%)	GAA (2.87%)	GAA (2.48%)	TGC (2.64%)	TTC (2.85%)	GAA (2.65%)	GCC (2.53%)	TTC (2.81%)	
5	TCA (2.48%)	TTT (2.61%)	TGC (2.61%)	TTC (2.46%)	GCA (2.61%)	TTT (2.53%)	TGC (2.50%)	GCA (2.49%)	TTT (2.72%)	
6	ATC (2.44%)	GCC (2.52%)	TCA (2.44%)	CAG (2.39%)	GGC (2.43%)	TCA (2.51%)	CAG (2.49%)	GCG (2.46%)	TCA (2.58%)	
7	ACG (2.29%)	TCA (2.40%)	ATC (2.26%)	TTT (2.31%)	TTC (2.33%)	TGC (2.46%)	ATC (2.45%)	TGC (2.23%)	GCC (2.43%)	
8	GAA (2.28%)	GAA (2.29%)	GCC (2.24%)	GGC (2.21%)	GCC (2.32%)	ATC (2.32%)	TCA (2.45%)	TCA (2.14%)	ATC (2.40%)	
9	GCC (2.26%)	GCG (2.19%)	TTT (2.24%)	TCA (2.21%)	CAG (2.17%)	GGC (2.19%)	GCC (2.30%)	CGC (2.13%)	GCG (2.31%)	
10	TTT (2.21%)	GTT (2.15%)	CAA (2.18%)	AGC (2.12%)	AGC (2.15%)	GCC (2.06%)	TTT (2.25%)	TTC (2.10%)	GTT (2.24%)	
	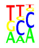	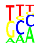	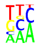	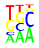	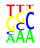	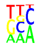	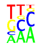	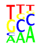	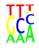	
-10	CCC (0.97%)	CTT (0.97%)	TGT (0.86%)	GTG (1.04%)	GGG (0.98%)	CCC (0.96%)	TGT (0.99%)	AGT (0.99%)	CCC (0.88%)	Least common
-9	GGA (0.97%)	GTA (0.94%)	AGG (0.85%)	CTC (1.02%)	AGT (0.97%)	CTT (0.91%)	TAT (0.99%)	GAG (0.93%)	CCT (0.87%)	
-8	CTC (0.95%)	ACT (0.94%)	ACT (0.85%)	GGA (0.99%)	AGG (0.96%)	GGG (0.89%)	ACT (0.98%)	GTA (0.88%)	CGA (0.82%)	
-7	AGA (0.91%)	CGA (0.88%)	GGG (0.85%)	ACT (0.96%)	CTC (0.93%)	AGT (0.84%)	GTA (0.97%)	CGA (0.82%)	CTT (0.80%)	
-6	GGG (0.90%)	AGT (0.82%)	AGT (0.84%)	GAG (0.94%)	GTA (0.92%)	AGG (0.80%)	AGG (0.96%)	AGA (0.80%)	ACT (0.80%)	
-5	AGT (0.89%)	GAG (0.76%)	CTT (0.82%)	AGT (0.92%)	CCT (0.91%)	CCT (0.77%)	GAG (0.91%)	CCT (0.71%)	GAG (0.78%)	
-4	AGG (0.78%)	GGA (0.75%)	AGA (0.82%)	GGG (0.88%)	GAG (0.82%)	GAG (0.74%)	AGA (0.84%)	ACT (0.70%)	AGA (0.64%)	
-3	GAG (0.75%)	AGA (0.70%)	GAG (0.64%)	AGG (0.80%)	ACT (0.80%)	ACT (0.65%)	GGA (0.78%)	GGA (0.63%)	GGA (0.61%)	
-2	TAG (0.41%)	TAG (0.49%)	CTA (0.35%)	CTA (0.55%)	CTA (0.55%)	CTA (0.47%)	TAG (0.48%)	CTA (0.60%)	TAG (0.46%)	
-1	CTA (0.41%)	CTA (0.44%)	TAG (0.33%)	TAG (0.44%)	TAG (0.46%)	TAG (0.35%)	CTA (0.47%)	TAG (0.55%)	CTA (0.44%)	
	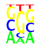	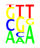	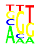	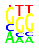	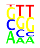	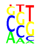	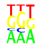	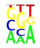	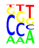	

Kmer space for 3-mers: 64 Random chance for any given 3-mer: 1.56%

4-mer error motif analysis

Rank	Template Insertion	Template Deletion	Template Substitution	Complement Insertion	Complement Deletion	Complement Substitution	2D Insertion	2D Deletion	2D Substitution	
1	ATCA (1.00%)	AAAA (1.04%)	GAAA (1.15%)	CAGC (1.00%)	CAGC (1.01%)	ATCA (0.99%)	ATCA (0.86%)	AAAA (1.73%)	GGCA (1.13%)	Most common
2	AACG (0.94%)	GAAA (0.99%)	TTTC (1.08%)	ATCA (0.90%)	CAAA (0.99%)	AGCA (0.98%)	GGCA (0.85%)	TTTT (1.61%)	GAAA (1.03%)	
3	TTTC (0.89%)	TGCC (0.99%)	AAAA (1.02%)	CTGC (0.87%)	AAAA (0.93%)	AAAA (0.96%)	GAAA (0.82%)	CAAA (1.58%)	AAAA (1.00%)	
4	GAAA (0.85%)	CAAA (0.91%)	GCAA (0.90%)	CAAA (0.81%)	GAAA (0.89%)	GAAA (0.95%)	CGCC (0.81%)	TAAA (1.28%)	CAAA (0.96%)	
5	TGCC (0.83%)	TTTC (0.90%)	ATCA (0.90%)	AGCA (0.77%)	ATTT (0.88%)	CAAA (0.95%)	CCAG (0.81%)	ATTT (1.25%)	TGAA (0.93%)	
6	CAGC (0.78%)	ATCA (0.89%)	GGCA (0.85%)	CGGC (0.76%)	CGGC (0.87%)	TGAA (0.92%)	TGCC (0.81%)	GAAA (1.09%)	AGCA (0.93%)	
7	CGCC (0.75%)	TTCA (0.87%)	AACG (0.83%)	TTGC (0.74%)	CTGC (0.85%)	AGAA (0.88%)	CAAA (0.79%)	CTTT (0.97%)	ATCA (0.90%)	
8	TTCA (0.75%)	CAGC (0.86%)	CAAA (0.83%)	CCAG (0.73%)	TGAA (0.84%)	TAAA (0.86%)	TGAA (0.78%)	GTTT (0.97%)	CGCA (0.89%)	
9	CAAA (0.74%)	TTTT (0.82%)	TGCC (0.82%)	TGGC (0.73%)	TAAA (0.84%)	GGCA (0.82%)	CAGC (0.77%)	CGCC (0.89%)	TGCA (0.88%)	
10	AAAA (0.74%)	CTGC (0.81%)	CTTC (0.82%)	AAAA (0.73%)	TGGC (0.81%)	GGAA (0.82%)	CTGC (0.74%)	TGCC (0.82%)	GGAA (0.87%)	
	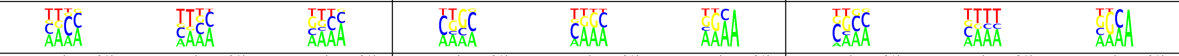									
-10	TAGT (0.11%)	CGAG (0.13%)	CTAT (0.10%)	TCTA (0.12%)	GGAC (0.12%)	GTAG (0.11%)	TATA (0.12%)	TACT (0.14%)	TCTA (0.11%)	Least common
-9	ACTA (0.10%)	GGAC (0.12%)	CGAG (0.10%)	TAGA (0.12%)	CTAT (0.12%)	GACT (0.11%)	CTAA (0.12%)	ACCT (0.14%)	ACTA (0.11%)	
-8	GGAC (0.10%)	CTAT (0.12%)	GGAC (0.09%)	TAGT (0.11%)	CCTC (0.12%)	TAGT (0.10%)	TAGT (0.12%)	GGGA (0.14%)	TATA (0.11%)	
-7	TATA (0.10%)	CTAA (0.12%)	TAGT (0.09%)	GGAC (0.11%)	TAGT (0.12%)	ACTA (0.10%)	CTAT (0.12%)	CGGA (0.14%)	CTAA (0.10%)	
-6	TTAG (0.10%)	TAGT (0.11%)	ACTA (0.09%)	TTAG (0.11%)	ACTA (0.11%)	TTAG (0.10%)	ACTA (0.11%)	CTAA (0.14%)	CTAT (0.09%)	
-5	TAGA (0.07%)	TCTA (0.10%)	TCTA (0.08%)	CCCT (0.10%)	TAGA (0.11%)	CTAT (0.10%)	TCTA (0.10%)	TAGG (0.11%)	CCCT (0.09%)	
-4	TCTA (0.07%)	TAGG (0.07%)	TAGA (0.07%)	CTAA (0.10%)	CCCT (0.10%)	CCCT (0.08%)	TAGA (0.08%)	CCCT (0.10%)	TAGG (0.07%)	
-3	TAGG (0.06%)	TAGA (0.06%)	TAGG (0.05%)	CCTA (0.07%)	TAGG (0.07%)	TAGG (0.06%)	TAGG (0.07%)	TAGA (0.08%)	TAGA (0.05%)	
-2	CCTA (0.04%)	CCTA (0.04%)	CCTA (0.04%)	TAGG (0.05%)	CCTA (0.06%)	CCTA (0.05%)	CCTA (0.06%)	CCTA (0.06%)	CCTA (0.04%)	
-1	CTAG (0.01%)	CTAG (0.01%)	CTAG (0.01%)	CTAG (0.02%)	CTAG (0.01%)	CTAG (0.01%)	CTAG (0.02%)	CTAG (0.01%)	CTAG (0.01%)	
	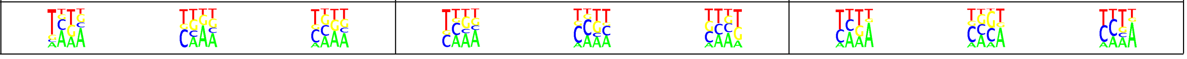									

Kmer space for 4-mers: 256   Random chance for any given 4-mer: 0.39%

5-mer error motif analysis

Rank	Template Insertion	Template Deletion	Template Substitution	Complement Insertion	Complement Deletion	Complement Substitution	2D Insertion	2D Deletion	2D Substitution	
1	CAGCA (0.39%)	CAGCA (0.41%)	CAGCA (0.44%)	CAGCA (0.46%)	CAGCA (0.42%)	CAGCA (0.57%)	CAGCA (0.42%)	ATTTT (0.61%)	CAGCA (0.50%)	Most common
2	CATCA (0.34%)	TTGCC (0.35%)	CATCA (0.33%)	TCAGC (0.31%)	ATAAA (0.33%)	ATAAA (0.38%)	CGGCA (0.33%)	GAAAA (0.59%)	CGGCA (0.44%)	
3	TTATC (0.31%)	GAAAA (0.34%)	GAAAA (0.33%)	CATCA (0.31%)	TCAGC (0.33%)	CATCA (0.37%)	GCAAA (0.29%)	GCAAA (0.56%)	GAAAA (0.35%)	
4	GCAAA (0.29%)	CATCA (0.34%)	ATTTC (0.31%)	CCAGC (0.30%)	GCTGC (0.32%)	CAGAA (0.35%)	TGGCA (0.27%)	TAAAA (0.51%)	GCAAA (0.35%)	
5	CAAAA (0.29%)	GCAAA (0.33%)	GCAAA (0.31%)	GCTGC (0.30%)	GAAAA (0.32%)	CGGCA (0.34%)	GCCAG (0.26%)	CAAAA (0.50%)	TGGCA (0.35%)	
6	GCGGC (0.28%)	CAAAA (0.33%)	CAAAA (0.31%)	GATGC (0.28%)	GCAAA (0.31%)	AAGAA (0.33%)	TTGCC (0.26%)	ATAAA (0.48%)	TTGCC (0.32%)	
7	TGCTG (0.28%)	ATTTT (0.32%)	AGAAA (0.30%)	GCGGC (0.28%)	CCAGC (0.31%)	TTATC (0.32%)	GCGCA (0.26%)	CTTTT (0.45%)	CGCCA (0.31%)	
8	CGTTT (0.28%)	GCTGC (0.29%)	TCTTC (0.29%)	ATAAA (0.28%)	ATTTT (0.30%)	AATCA (0.30%)	CATCA (0.25%)	GTTTT (0.42%)	TGAAA (0.30%)	
9	TTGCC (0.28%)	ATAAA (0.29%)	TGAAA (0.29%)	CTGGC (0.27%)	AAGAA (0.29%)	GCTGC (0.30%)	CGCCA (0.25%)	ACAAA (0.41%)	CATCA (0.30%)	
10	CGCCA (0.28%)	CTGCC (0.29%)	CGTTC (0.29%)	GCAGC (0.26%)	ACAAA (0.29%)	AGAAA (0.29%)	GCTGG (0.25%)	GATTT (0.37%)	ATAAA (0.30%)	
	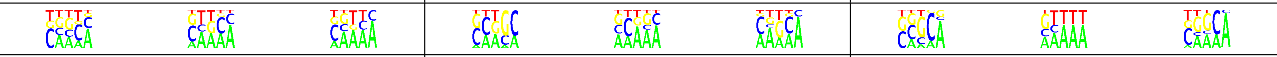									
-10	TAGGG (0.01%)	TAGAT (0.01%)	ACCTA (0.01%)	GGACC (0.01%)	TCCTA (0.01%)	CTTAG (0.01%)	CCCTA (0.01%)	CTAGC (0.01%)	TTGGA (0.01%)	Least common
-9	TCCTA (0.01%)	CCCTA (0.01%)	CCCTA (0.00%)	CCTAT (0.01%)	TAGGT (0.01%)	CTAGC (0.01%)	GCTAG (0.01%)	TAGGA (0.01%)	ACCTA (0.01%)	
-8	CTTAG (0.01%)	CTAGT (0.00%)	GCTAG (0.00%)	ACCTA (0.01%)	CTAGC (0.01%)	GCTAG (0.01%)	CTAGC (0.01%)	TCCTA (0.01%)	CTAGC (0.00%)	
-7	CCCTA (0.01%)	CTAGC (0.00%)	CTAGG (0.00%)	CCCTA (0.01%)	GCTAG (0.01%)	TAGGT (0.01%)	TAGGA (0.01%)	GCTAG (0.01%)	GCTAG (0.00%)	
-6	GGACC (0.00%)	GCTAG (0.00%)	ACTAG (0.00%)	CTAGA (0.00%)	CTAGT (0.00%)	CTAGT (0.00%)	CTAGA (0.00%)	ACTAG (0.01%)	CTAGT (0.00%)	
-5	CTTGG (0.00%)	ACTAG (0.00%)	CTAGT (0.00%)	CCTAG (0.00%)	CTAGA (0.00%)	ACTAG (0.00%)	CTAGT (0.00%)	CTAGT (0.00%)	CTAGG (0.00%)	
-4	ACTAG (0.00%)	CTAGG (0.00%)	CTAGC (0.00%)	CTAGT (0.00%)	TCTAG (0.00%)	CTAGG (0.00%)	ACTAG (0.00%)	CTAGA (0.00%)	ACTAG (0.00%)	
-3	GCTAG (0.00%)	TCTAG (0.00%)	TCTAG (0.00%)	CTAGG (0.00%)	CTAGG (0.00%)	CTAGA (0.00%)	TCTAG (0.00%)	TCTAG (0.00%)	CTAGA (0.00%)	
-2	CTAGT (0.00%)	CTAGA (0.00%)	CCTAG (0.00%)	TCTAG (0.00%)	CCTAG (0.00%)	TCTAG (0.00%)	CTAGG (0.00%)	CTAGG (0.00%)	TCTAG (0.00%)	
-1	CTAGA (0.00%)	CCTAG (0.00%)	CTAGA (0.00%)	ACTAG (0.00%)	ACTAG (0.00%)	CCTAG (0.00%)	CCTAG (0.00%)	CCTAG (0.00%)	CCTAG (0.00%)	
	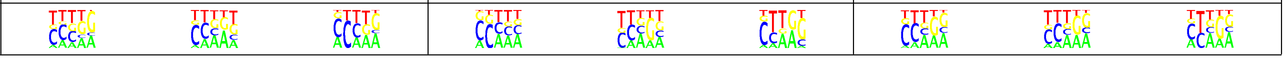									

Kmer space for 5-mers: 1024   Random chance for any given 5-mer: 0.10%
